# Loop-Mediated Isothermal Amplification Assay for Detection of Generic and Verocytotoxin-Producing *Escherichia coli* among Indigenous Individuals in Malaysia

**DOI:** 10.1155/2014/457839

**Published:** 2014-05-19

**Authors:** Cindy Shuan Ju Teh, Kek Heng Chua, Yvonne Ai Lian Lim, Soo Ching Lee, Kwai Lin Thong

**Affiliations:** ^1^Department of Medical Microbiology, Faculty of Medicine, University of Malaya, 50603 Kuala Lumpur, Malaysia; ^2^Department of Biomedical Science, Faculty of Medicine, University of Malaya, 50603 Kuala Lumpur, Malaysia; ^3^Department of Parasitology, Faculty of Medicine, University of Malaya, 50603 Kuala Lumpur, Malaysia; ^4^Institute of Biological Sciences, Faculty of Science, University of Malaya, 50603 Kuala Lumpur, Malaysia

## Abstract

We have successfully developed a Loop-mediated isothermal amplification ** (**LAMP) assay that could specifically detect generic *Escherichia coli* (*E. coli*). This assay was tested on 85 bacterial strains and successfully identified 54 *E. coli* strains (average threshold time, Tt = 21.26). The sensitivity of this assay was evaluated on serial dilutions of bacterial cultures and spiked faeces. The assay could detect 10^2^ CFU/mL for bacterial culture with Tt = 33.30 while the detection limit for spiked faeces was 10^3^ CFU/mL (Tt = 31.12). We have also detected 46 generic *E. coli* from 50 faecal samples obtained from indigenous individuals with 16% of the positive samples being verocytotoxin-producing *E. coli* (VTEC) positive. VT1/VT2 allele was present in one faecal sample while the ratio of VT1 to VT2 was 6 : 1. Overall, our study had demonstrated high risk of VTEC infection among the indigenous community and most of the asymptomatic infection occurred among those aged below 15 years. The role of asymptomatic human carriers as a source of dissemination should not be underestimated. Large scale screening of the VTEC infection among indigenous populations and the potential contamination sources will be possible and easy with the aid of this newly developed rapid and simple LAMP assay.

## 1. Introduction

The human body is inhabited by a vast number of microorganisms. It has been reported that there are 10^14^ bacterial cells present in our body [1]. Besides fulfilling their physiological functions, the microbiota has a direct impact on human health [2]. Basically, the microbiota plays important roles in combating aggression from other microorganisms [3] as well as maintaining the balance of the intestinal mucosa and providing an innate defence [4].

*Escherichia coli* (*E. coli*) is an inhabitant of the intestines and faeces of humans and animals. It is among the first bacterial species to colonize the intestine during infancy. Commensal* E. coli* strains found mainly in the caecum and the colon provide some benefits to their host by inducing colonization resistance in the host through the production of bacteriocins and through other mechanisms [5]. Most of the* E. coli* strains are harmless,* but they can* also cause extraintestinal* E. coli* (ExPEC) infections in urinary tract, meninges, and bloodstream.

Nevertheless, there are certain serotypes that can cause diseases in humans and animals [6, 7]. Verocytotoxin-producing* Escherichia coli* (VTEC) is a serious public health concern as it has been responsible for outbreaks in European countries [8, 9] and has been associated with haemolytic-uraemic syndrome (HUS), the leading cause of acute renal failure in children [7, 10]. On the other hand, VTEC could also colonize the human intestine without causing any symptoms [6, 11]. This infection might not be harmful to the host but remains a threat to the public as asymptomatic carriers are the potential source for transmission of VTEC; hence, prompt detection of carrier is important.

Given that instantaneous detection of the infectious agents is important, hence molecular approaches have been utilized as the detection is more rapid than conventional biochemical tests. Compared to PCR, loop-mediated isothermal amplification (LAMP) produces more specific results as there are 4 primers targeting 6 regions within a gene. Besides, LAMP is also more rapid (reaction occurs at constant temperature) and eliminates the need for electrophoresis as the results can be monitored through real-time turbidimeter or visually based on turbidity or with the aid of SYBR I dye [12].

In Malaysia, studies on sanitation-related diseases such as parasitic infections are commonly carried out among the indigenous communities due to their poor living conditions, inappropriate hygiene practices, and the lack of functioning toilet facilities in their houses [13]. Asymptomatic parasitic infections among indigenous individuals have been reported [14, 15]; however, the study on bacterial infections is relatively scarce. Previously, Rahim et al. [16] had conducted a study on the prevalence of* Helicobacter pylori* (HP) infection among the indigenous individuals. A total of 19% asymptomatic indigenous individuals were tested positive for the HP infection. The limited amount of information regarding bacterial infections may have been contributed to the lack of rapid technique which has the potential to be applied in the field. This is crucial as many of the indigenous communities are situated in remote areas which is logistically challenging if samples were to be processed within a few hours. Development of cost-effective and rapid modern tools which have the potential to overcome these limitations will be crucial.

In the present study, we aim to develop a LAMP assay that can specifically detect generic* E. coli*. The developed assay will be applied in combination with another VTEC-specific LAMP assay previously developed by Hara-Kudo et al. [17] to detect the presence of* E. coli* and VTEC among Orang Asli community in Malaysia.

## 2. Materials and Methods

### 2.1. Bacteria Strains

Fifty* E. coli* strains (5 verocytotoxin-producing and 45 nonverocytotoxin-producing* E. coli*) and 35 bacterial species other than* E. coli* (including 10* Shigella* spp.) were revived from the culture collections in the Laboratory of Biomedical Science and Molecular Microbiology, Institute of Graduate Studies, University of Malaya. A loopful of colonies of overnight culture was suspended in 100 *μ*L ddH_2_O and was subjected to boiling at 99°C for 5 min. The suspension was snapped cool and centrifuged. The supernatant was quantified and 1.5 *μ*L (equivalent to ~50 ng) was used as the DNA template for LAMP assay and PCR.

### 2.2. LAMP Assay

mGenomeSubtractor [18] and web-based Artemis Comparison Tool (WebACT) (http://www.webact.org/WebACT/) were used to compare the published genomes of* E. coli *and other Enterobacteriaceae, such as* Shigella *spp.,* Salmonella*,* Yersinia* spp., and* Klebsiella* spp. A list of conserved regions for* E. coli* was generated and was again blasted against the database by using NCBI BLAST (http://blast.ncbi.nlm.nih.gov/Blast.cgi). A CDS encoding glycerate kinase (EcolC_3109, Accession number: CP000946) was selected and used for primers design. Two pairs of primers, including F3 (5′-GGTAGATCGAACGGTCATCG-3′), B3 (5′-GGCCAGCAACGGATTACG-3′), forward inner primer (FIP) (5′-CGCAGACTTCAAGCGTCACGATCGAAGGAACGGTGGATGC-3′), and backward inner primer (BIP) (5′-CCTTACCGGCGACGGGAAAA-CTTTTCAGGCGCGACCAG-3′) as well as a loop primer (LB) (5′-TGAGATGGCGGCAGCAAGTG-3′) were designed by using online PrimerExplorer V4 program (PrimerExplorer, Eiken Chemical Co., Ltd.).

The LAMP assay was optimized on* E. coli* strains using Loopamp DNA amplification kit (Eiken Chemical Co., Ltd., Tokyo, Japan). Briefly, the reaction was carried out in a total of 25 *μ*L containing 40 pmol of each FIP and BIP primers, 5 pmol of each F3 and B3 primers, 10 pmol of LB primer, 12.5 *μ*L of 2X reaction mixture, 1 *μ*L of Bst DNA polymerase, and 1.5 *μ*L of DNA (approximately 100 ng). The reaction mixtures were incubated at 62°C for 80 min, followed by enzyme inactivation at 80°C for 2 min in the Loopamp real-time turbidimeter (LA-320, Teramecs Co., Ltd., Kyoto, Japan). The positive result was indicated when the turbidity reached 0.1 within 60 min at 65 nM and Tt was recorded.

### 2.3. PCR Validation

PCR was performed in parallel to validate the results produced by LAMP assay. Briefly, PCR targeting* phoA* gene for* E. coli* detection and VT1 or VT2 gene for verocytotoxin producer was carried out as described by Ho et al. [19].

### 2.4. Detection Limit of LAMP Assay on Bacterial Culture and Spiked Faeces

Overnight cultures of* E. coli* were serially diluted 10-fold. An aliquot of 100 *μ*L of each dilution was used for the DNA template preparation and plated on lysogeny agar for CFU count. On the other hand, the overnight culture was also spiked in 900 *μ*L of stools (diluted with brain-heart infusion broth to avoid inhibition during PCR), followed by a 10-fold serial dilution. An aliquot of 100 *μ*L of the dilution was plated on the agar for viable count while another 100 *μ*L was used for the extraction of DNA by using crude lysate method. The DNA templates were subjected to LAMP assay.

### 2.5. Field Evaluation of LAMP Assay for the Detection of VTEC among Indigenous Individuals

A total of 50 indigenous individuals with positive parasitic infections were recruited in the study (Ethics committee/IRB reference number: 824.11). Sample collection procedure has also been approved by the Department of Orang Asli Development (JAKOA). Consent was given by the participating indigenous individuals either via signature or via thumbprint prior to sample collection. Faecal samples were subjected to DNA extraction using QIAamp DNA Stool Mini Kit (QIAGEN, The Netherlands). The extracted total DNA was quantified and stored at −20°C until further use.

The LAMP assay developed in this study was used to detect generic strains of* E. coli* that are present in the faecal sample while VT1 and VT2 producer were determined by using LAMP assay that was previously described by Hara-Kudo et al. [17]. The reaction consisted of approximately 200–400 ng total DNA incubated at 62°C for 45 min and the positive reactions were detected based on the colour change after the addition of a SYBR green 1 : 10 dilution to the reaction tube.

## 3. Results

**Figure 1:**
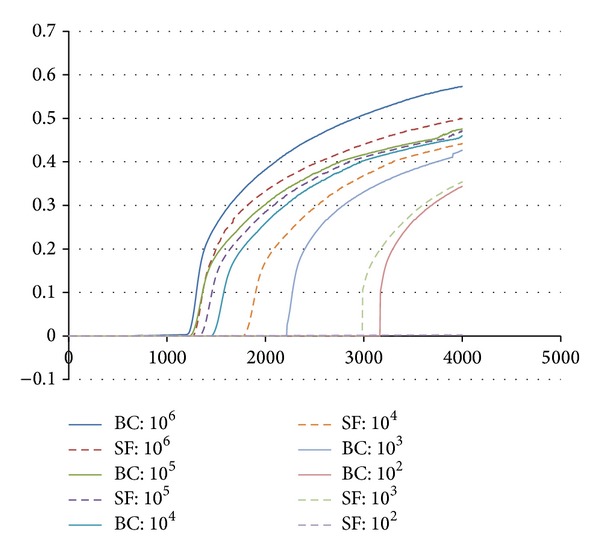
Sensitivity of LAMP assay of bacterial culture (BC) and spiked faecal (SF) specimens on different CFU (from 10^6^ to 10^2^).

All the 50* E. coli* strains showed positive reaction within 25 min with an average Tt value of 21.26. None of the non-*E. coli* strains showed positive amplification. This indicates the high specificity of the LAMP assay for* E. coli* detection. Furthermore, the detection limit of the LAMP assay was as low as 10^2^ CFU/mL and 10^3^ CFU/mL on bacterial culture (Tt = 33.30) and the spiked faeces (Tt = 31.12), respectively. For bacterial culture, the reaction with more than 10^5^ CFU/mL of cells yielded positive results within 18 min, while, for faecal samples, only those reactions which have more than 10^6^ CFU/mL of cells showed positive amplification before 18 min (Figure 1).

The usefulness of the newly developed* E. coli* LAMP assay was evaluated using the faecal samples collected from Orang Asli. Overall, the results obtained by using LAMP assay were in concordance with PCR except for 1 sample (Table 1). To verify the result of LAMP assay, the amplicon of F3 and B3 of this particular sample was sent for sequencing. The amplicon was 100% matched with the* E. coli* glycerate kinase gene segment.

On the other hand, all* E. coli* positive samples were also subjected to VT1 and VT2 detection as described in Ho et al. [19]. Among the 46* E. coli* positive samples, VT1 was detected in 6 volunteers (≥15 years old) while VT2 was detected in one adult's sample. Only one sample obtained from a 5-year-old girl was detected with VT1 and VT2 producing* E. coli*.

## 4. Discussion

In this study, a LAMP assay targeting glycerate kinase of* E. coli* was developed. The assay demonstrated a high specificity and was fairly sensitive when applied on bacterial culture (10^2^) and spiked faecal sample (10^3^). None of the* Shigella *spp. showed positive reaction and this indicated that the developed LAMP assay could specifically differentiate* E. coli* from* Shigella*. Therefore, this assay could be potentially applied on food and animal samples.

LAMP assays targeting different strains/pathotypes of* E. coli* have been described previously [17, 20–22]. Among the published studies, Hill et al. [21] had developed a LAMP assay targeting common strains of* E. coli* and this assay was evaluated using urine samples.* malB* gene was used for the primer design; however, this gene was also found in* Shigella*. The developed assay worked well in the identification of urinary* E. coli* (as* Shigella* is rare in extraintestinal infection) but will be restricted to other applications such as detection of common* E. coli* strains from faecal, food, soil, and water samples.

*E. coli* and* Shigella* are closely related. The validity of* Shigella *genus has become a contentious issue over the past decades. Based on the conventional view,* Shigella *and* E. coli *shared greater than 90% homology by DNA-DNA reassociation analysis and* Shigella *are* E. coli* strains that undergo the convergent evolution of* Shigella* phenotypic properties that contribute to the* Shigella* pathotype [23–25]. However, in terms of clinical manifestation,* Shigella* spp. are frank pathogens that readily cause disease in humans while* E. coli* normally lives in the intestines of humans and animals (with the exception of pathogenic clones). Prompt and reliable identification of these infectious agents is important for the right choice of treatment and to avoid any complications.

Differentiation between* E. coli* and* Shigella* is difficult but achievable based on physiological, biochemical, and serological typing [26]. For a more rapid method, alkaline phosphatase (*phoA*) gene has been used in PCRs for common* E. coli* strains detection, demonstrating high specificity [27, 28]. Therefore, this PCR was selected as a tool for validation of the results obtained by using LAMP in this study. In fact, the LAMP assay showed higher specificity in detecting generic* E. coli* in the indigenous samples compared to PCR targeting* phoA *gene, where the PCR could not detect* E. coli* in one of the samples (PG3.1).

The rate of verocytotoxin-producing* E. coli* in asymptomatic human carrier in this study was considered high (16%) compared to previous studies that reported the rate of 7.6% and 4%, conducted by Hong et al. [6] and Stephan and Hoelzle [11], respectively. In the studies of Hong et al. [6] and Stephan and Hoelzle [11], workers in slaughterhouse were targeted as they were considered at higher risk for carriage or excretion of VTEC. Thus, the finding in this study has highlighted the attention on hygiene practices and living conditions of the indigenous populations in Malaysia, given the fact that these communities are afflicted with parasitic infections not only as reported previously [14, 15], but also as a reservoir for VTEC.

In this study, all the volunteers were infected by different parasites such as* Giardia*,* Trichuris*,* Ascaris*, Hookworm,* Entamoeba coli,* and* Iodamoeba*. There is no statistical evidence that showed correlation between parasitic infection and VTEC colonization. Our results also did not suggest any groups (single or multiple parasitic infections) that are prone to VTEC colonization. Interestingly, we found that* E. coli* was hardly detected in the stool samples containing multiple parasites as in the first run of LAMP assay (sample PM 25.2, DB 2.1, BP 1.5, BP 14.2, DK 17.3, DK 17.6, DK 19.10, DK 27.1, UK 15.5, UL 13.2, NT 6.2, NT 6.3, and SW 16.3). Reasons that influenced the sensitivity of this LAMP assay will be further investigated. Meanwhile, the 4 samples which were not detected with* E. coli* might be due to recent antibiotic treatments.

## 5. Conclusion

We have successfully developed a LAMP assay that can specifically detect generic* E. coli* and further performed in combination with the LAMP assay that detect verocytotoxin-related gene. Our study suggests the high risk of indigenous community for carriage and excretion of VTEC. Due to the high specificity and sensitivity of the generic* E. coli* LAMP assay developed in this study, we proposed the application of this assay in food and animals as an alternative approach for rapid and more specific detection of* E. coli*.

## Figures and Tables

**Table 1:** PCR and LAMP results for the samples obtained from indigenous individuals which were previously detected with different parasitic infections.

ID	AGE	Parasitic infection	PCR	LAMP (*E. coli*)	
				Generic *E. coli *	VT producer
PG 12.2	8	*Giardia *	+	+	
PG 20.1	12	*Giardia, Entamoeba coli *	−	−	
PG 3.1	10	*Giardia *	−	+	
PB 11.1	73	*Giardia *	+	+	
PB 14.5	5	*Giardia *	+	+	VT1/VT2
PM 19.2	7	*Giardia *	+	+	
PM 25.2	7	*Giardia, Entamoeba coli *	+	+	
PM 31.2	8	*Giardia *	+	+	
PM 53.1	10	*Giardia *	−	−	
PM 53.2	9	*Giardia *	+	+	
PM 54.1	10	*Giardia *	+	+	
SP 11.1	6	*Giardia *	+	+	
SP 7.3	1	*Giardia *	+	+	
DB 11.7	15	*Giardia *	+	+	VT1
DB 2.1	30	*Giardia, Trichuris, Ascaris, Entamoeba coli, Iodamoeba *	+	+	
BP 1.5	4	*Giardia, Trichuris *	+	+	
BP 14.2	10	*Giardia, Trichuris, Ascaris *	+	+	
SB 3.3	11	*Giardia *	+	+	VT1
DK 10.4	7	*Giardia, Trichuris *	+	+	
DK 17.3	6	*Giardia, Trichuris, *Hookworm*, Entamoeba coli *	+	+	
DK 17.6	13	*Giardia, Trichuris, Entamoeba coli *	+	+	
DK 19.10	8	*Giardia, Trichuris, Ascaris *	+	+	
DK 27.1	10	*Giardia, Trichuris *	+	+	
DK 29.1	7	*Giardia, Trichuris *	+	+	
UK 15.2	32	*Giardia *	+	+	
UK 15.5	4	*Giardia, Trichuris, Ascaris *	+	+	
UK 17.3	7	*Giardia, Ascaris *	+	+	
UK 6.2	45	*Giardia, Ascaris *	+	+	
UK 6.5	13	*Giardia, Ascaris *	+	+	VT1
UL 13.2	5	*Giardia, Trichuris, Ascaris *	+	+	VT1
UL 3.3	12	*Giardia, Trichuris, Ascaris, *Hookworm	−	−	
UL 4.4	1	*Giardia *	+	+	
UK 15.3	9	*Giardia, Ascaris *	+	+	
UK 17.1	32	*Giardia, Ascaris *	−	−	
UK 6.3	18	*Giardia *	+	+	
PP 1.1	58	*Giardia, Trichuris, Ascaris *	+	+	
PS 7.3	11	*Giardia *	+	+	
NT 4.4	13	*Giardia, Trichuris, Ascaris *	+	+	
NT 6.2	30	*Giardia, Ascaris *	+	+	VT2
NT 6.3	8	*Giardia, Ascaris, Entamoeba. coli *	+	+	
PG 2.7	17	*Giardia, *Hookworm	+	+	
PG 26.2	43	*Giardia *	+	+	
JJ 7.2	36	*Giardia *	+	+	
JJ 11.4	7	*Giardia *	+	+	VT1
JJ 11.5	5	*Giardia *	+	+	VT1
SW 5.3	10	*Giardia *	+	+	
SW 10.1	31	*Giardia *	+	+	
SW 11.2	1	*Giardia *	+	+	
SW 14.3	4	*Giardia, E. coli *	+	+	
SW 16.3	32	*Giardia, *Hookworm	+	+	
